# Release of IL-1**β** Triggered by Milan Summer PM_10_: Molecular Pathways Involved in the Cytokine Release

**DOI:** 10.1155/2013/158093

**Published:** 2013-02-06

**Authors:** Rossella Bengalli, Elisabetta Molteni, Eleonora Longhin, Magne Refsnes, Marina Camatini, Maurizio Gualtieri

**Affiliations:** ^1^Polaris Research Centre, Department of Environmental Sciences and Earth Sciences, University of Milano-Bicocca, Piazza della Scienza 1, 20126 Milano, Italy; ^2^Department of Biology and Biotecnology, University of Pavia, Via Ferrata 1, 27100 Pavia, Italy; ^3^Division of Environmental Medicine, Norwegian Institute of Public Health, P.O. Box 4404, Nydalen, 0403 Oslo, Norway; ^4^UTTS Saluggia, ENEA, Strada Crescentino, 13040 Saluggia, Italy

## Abstract

Particulate matter (PM) exposure is related to pulmonary and cardiovascular diseases, with increased inflammatory status. The release of the proinflammatory interleukin- (IL-) 1**β**, is controlled by a dual pathway, the formation of inactive pro-IL-1**β**, through Toll-like receptors (TLRs) activation, and its cleavage by NLRP3 inflammasome. THP-1-derived macrophages were exposed for 6 h to 2.5 **μ**g/cm^2^ of Milan PM_10_, and the potential to promote IL-1**β** release by binding TLRs and activating NLRP3 has been examined. Summer PM_10_, induced a marked IL-1**β** response in the absence of LPS priming (50-fold increase compared to unexposed cells), which was reduced by caspase-1 inhibition (91% of inhibition respect summer PM_10_-treated cells) and by TLR-2 and TLR-4 inhibitors (66% and 53% of inhibition, resp.). Furthermore, summer PM_10_ increased the number of early endosomes, and oxidative stress inhibition nearly abolished PM_10_-induced IL-1**β** response (90% of inhibition). These findings suggest that summer PM_10_ contains constituents both related to the activation of membrane TLRs and activation of the inflammasome NLPR3 and that TLRs activation is of pivotal importance for the magnitude of the response. ROS formation seems important for PM_10_-induced IL-1**β** response, but further investigations are needed to elucidate the molecular pathway by which this effect is mediated.

## 1. Introduction

In the last decade great effort has been paid to understand the mechanisms involved in particulate matter (PM) induced adverse health effects. Epidemiological evidence shows an association between exposure to air pollution and the occurrence of respiratory pathologies (chronic bronchitis, COPD) and exacerbation of allergic conditions such as asthma [1–3]. Furthermore, many studies also show an association between PM atherothrombotic effects, cardiovascular morbidity, and mortality [4–6]. 

PM is a heterogeneous pollutant composed of particles of different chemical composition and different sizes (defined as PM_10_, PM_2.5_, and PM_0.1_ for their aerodynamic diameter). Although the size determines the site of deposition of PM in the respiratory tract [7], the chemical composition of the inhaled particles is considered of primary importance in determining the adverse biological effects [8, 9]. The chemical properties of PM are strongly related to the sources of emission of the particles, and this is known to be crucial for the differences of the PM effects from different sampling sites [10–13].

The fine fraction (PM_2.5_) is generally composed of primary particles derived from combustion processes, mostly consisting of primary particles with mean diameter lower than 100 nm (PM_0.1_, ultrafine particles) and secondary aerosol deriving from chemical reaction of free compounds in the atmosphere. The particle composition reflects the sources of emission; indeed fine PM has usually higher content in organic compounds (such as PAHs) and elemental carbon (the soot core of the particles) than the coarse PM. 

The coarse fraction (PM_10–2.5_) is on the contrary dominated by particles derived from abrasion processes, such as the erosion of crustal material, resuspension of deposited particles, and biological components. We have previously shown that the season of PM sampling strongly influences the chemical and biological composition of both coarse and fine PMs [14, 15]. In fact summer and winter PM_10_ fractions showed a completely different composition in chemical and biological constituents, the latter being higher in summer PM_10_ [11, 16, 17]. 

Moreover, the chemical characterization showed that the PM_10_ contained crystal silica and other elements which can contribute to its inflammatory potential. 

A lot of studies have shown that PM_10_ exposure promotes inflammation in the lung which is associated with a systemic inflammatory response. Macrophages and lung epithelial cells incubated with PM_10_ release significantly increased amounts of cytokines and chemokines, including granulocyte-macrophage colony-stimulating factor (GM-CSF), interleukin IL-1*β*, IL-6, and IL-8, and macrophage chemo-attractant protein (MCP)-1 [18]. An increased lung inflammation is known to be fundamental for the development of different lung diseases, such as COPD [19–21]. However, despite the increased evidence that the coarse fraction of PM is potent in inducing lung inflammation, a model explaining its effects has not been completely understood. 

A critical property of the innate immune system is its ability to discriminate microbes from “self” by the recognition of invariant microbial structure called pathogen-associated recognition patterns (PAMPs) such as lipopolysaccharides (LPS) [22]. The sensing of these PAMPs is usually mediated by the membrane-bounded Toll-like receptors (TLRs), such as TLR-2 and TLR-4 [23, 24]. Commonly these receptors trigger the activation of the NF-kB pathway which determines the release of different proinflammatory proteins, such as pro-IL-1*β*. Another set of pattern recognition receptors are the cytoplasm Nod-like receptors. These receptors have been demonstrated to be key proteins in the activation of pro-caspase-1, through the formation of the caspase-1 activating platforms, the inflammasomes. The inflammasomes control in turn the cleavage and secretion of potent proinflammatory interleukins such as IL-1*β* and IL-18. Among the different inflammasomes, the NLRP3 (or NALP3) is the most characterized. This complex is composed of a basic scaffold, the adaptor molecule apoptosis-associated speck-like protein containing a caspase recruitment domain (ASC), and the caspase-1. The activation of this complex has been related to the exposure of different PAMPs as well as host-derived molecules [25]. 

IL-1*β* is released at the site of injury, or immunological challenge is coordinating inflammatory responses, such as the recruitment of other cells to the site of infection or injury [26], and is known to be crucial in development of different diseases, including silicosis [27, 28]. IL-1*β* is also, however, known to regulate sleep, appetite, and body temperature. Due to its potent activities, it is not surprisingly that IL-1*β* activity is rigorously controlled throughout its entire release pathway, from expression to maturation and final secretion. 

The activation of the inflammasome machinery has been related to different mechanisms which have been reviewed in [25]. However for the release of IL-1*β* a priming stimulus is required for the formation of pro-IL-1*β* as reported in [29].

It has been shown that particles occurring in ambient PM, such as crystalline silica, as well as different nanoparticles, may induce inflammasome activation [20, 30–33]. The potential role of the inflammasome in PM-induced inflammation is however not known. Reactive oxygen substances (ROS) are known to be involved in PM_10_-induced inflammation [34, 35] and also in silica-induced inflammasome activation [21, 36]. Potentially ROS might be involved in the pro-IL-1*β* formation as well as the inflammasome activation [37, 38].

In the present study it was hypothesized that PM_10_ due to its chemical and physical nature might induce IL-1*β* release. Summer Milan PM_10_ contains both endotoxins, which might activate TLR receptors, and elemental and crustal constituents, which might activate the inflammasome mechanism. Furthermore, it is hypothesized that ROS is involved in PM_10_-induced IL-1*β* responses.

## 2. Materials and Methods

### 2.1. Cell Culture and Treatments

The human monocytes cell line, THP-1, was maintained in Opti-MEM medium supplemented with 10% FBS and 100 U/100 mL Penicillin/Streptomycin at 37°C, 5% CO_2_. THP-1 cells were differentiated into macrophage-like cells by incubation with phorbol myristate acetate (PMA, 20 nM) (Sigma Aldrich) for 24 h. PMA was then removed and cells were washed and incubated in Opti-MEM (Invitrogen, Italy) medium supplemented with 20% FBS o/n. Cells were treated in 10% FBS medium with summer PM_10_ at different concentrations (1 *μ*g/cm^2^, 2.5 *μ*g/cm^2^, and 5 *μ*g/cm^2^) for different times of exposure (30 min, 2 h, 4 h, and 6 h). Winter PM_10_ and carbon black (CB, 2–12 *μ*m, Sigma Aldrich, Italy) were used (5 *μ*g/cm^2^) as comparison and reference particles, respectively. In order to investigate TLR-2, TLR-4 and caspase-1 involvement in IL-1*β* release, cells were pre-treated for 1 h with inhibitors of TLR-2 and TLR-4 (0, 1 *μ*g/mL, R&D Systems) and caspase-1 (z-YVAD-fmk, 10 *μ*M, Calbiochem) and then exposed to summer PM_10_ (2.5 *μ*g/cm^2^). Summer and winter PM_10_ particles have been characterised as previously reported in [39] and [40] respectively. ROS involvement in IL-1*β* release was assessed by treating PM_10_ exposed cells with N-acetylcysteine (NAC, 15 mM, 30 min prior to PM exposure).

### 2.2. IL-1*β* Release

Supernatants from control and PM_10_-exposed cells were collected and stored at −80°C. Supernatants were assayed for IL-1*β* with ELISA kits (Invitrogen Srl) according to manufacturer's instructions.

### 2.3. Endocytic Pathway Analysis

#### 2.3.1. Immunostaining

 After 30 min exposure to summer PM_10_, cells were washed in phosphate-buffered saline 1X (PBS), fixed in paraformaldehyde 4% for 20 min, and washed twice in PBS. Fixed cells were permeabilized with 0.1% Triton X-100 (Sigma Aldrich), 0.1% Tween (Sigma Aldrich), and 2% BSA (Sigma Aldrich) in PBS and incubated o/n with the rabbit anti-human early endosome antibody 1 (EEA1 Antibody, Cell Signaling Technology; dilution 1 : 100). Cells were then washed in PBS and incubated with Alexa fluor-488 (Invitrogen Molecular Probes Srl; dilution 1 : 1000) for 2 h. Samples were mounted on a slide with ProLong mount (Invitrogen Srl) and observed by Axio Observer inverted microscope (Zeiss, Germany).

#### 2.3.2. Western Blot

After exposure to summer PM_10_ cells were washed in PBS and stored at −80°C. Cells were then lysed in RIPA buffer (50 mM Tris-HCl pH 8; 150 mM NaCl; 1% NP-40; 0.5% sodium deoxycholate; 0.1% SDS; Sigma Ladrich Italy) and then sonicated three times for 30 sec on ice. Cell lysates were then separated by 8% SDS-PAGE and transferred on nitrocellulose membranes. Blots were incubated with rabbit polyclonal antibody against human EEA1 (Cell Signaling Technology; dilution 1 : 1000) o/n or anti-actin antibody (Sigma Aldrich, Italy; dilution 1 : 2000). After washes, the membranes were incubated with secondary antibody anti-rabbit IgG (Fab2 fragment-Alkaline Phosphatase, Sigma Aldrich; dilution 1 : 10000) and subsequently incubated with SIGMA FAST BCIP/NBT alkaline phosphatase substrate (Sigma Aldrich) for 10 min for detection. Fold increase data over control, obtained by acquisition of membrane and densitometry analysis with dedicated software (UVP, US), were normalized to the actin content.

### 2.4. Cells-Particles Interaction

#### 2.4.1. Haematoxylin-Eosin Staining

THP-1-derived macro-phage untreated and treated with summer PM_10_ for 24 h at the concentration of 2.5 *μ*g/cm^2^, were fixed in paraformaldehyde 4% for 20 min and then stained following haematoxylin-eosin protocol and then observed under an Axiolab light microscope (Zeiss, Germany).

#### 2.4.2. Transmission Electron Microscopy

Samples were prepared for transmission electron microscopy (TEM) using standard procedures. At the end of exposure the cells were fixed in 2.5% glutaraldehyde for 20 min at 4°C and postfixed with 1% osmium tetroxide for 1 h, followed by dehydration using a scale of graded ethanol. Cells were then embedded in Epon resin, and semithin and ultrathin sections were prepared by an ultramicrotome (Ultracut Jung E, Reichert Germany). Ultrathin slides were mounted on copper grids and counterstained by lead citrate and uranyl acetate prior to examination by Jeol JEM 1220 microscope operating at 80 kV and digital images were taken with a Gatan CCD camera.

### 2.5. Statistical Analysis

Results are reported as mean ± standard deviation of at least three independent experiments. Statistical differences were analysed by the software SigmaStat 3.1 performing ANOVA test with post hoc analysis (Dunn's); if required a parametric statistical analysis was performed.

## 3. Results

### 3.1. Release of IL-1*β* from Human Macrophage-Like Cell after Summer Milan PM_10_ Exposure

**Figure 1:**
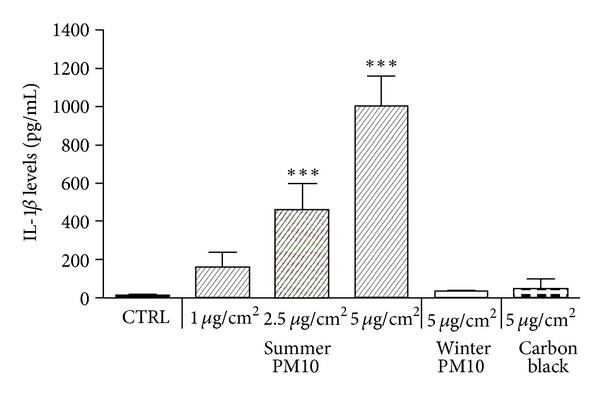
IL-1*β* release by THP-1-derived macrophages exposed for 6 h to different PM_10_ and carbon black (CB). The cells were exposed to increasing concentrations of summer Milan PM_10_, and one concentration (5 *μ*g/cm^2^) of winter PM_10_ and CB. CTRL: unexposed cells. Results are the mean and s.d. of three independent experiments and presented as pg/mL released in the culture medium. ***P < 0.001 versus CTRL.

**Figure 2:**
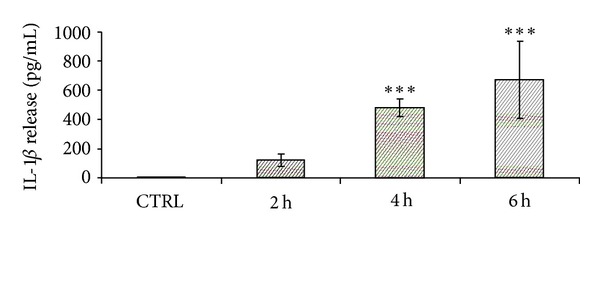
Time-dependent release of IL-1*β* in THP-1-derived macrophages cells treated for 2, 4, and 6 h with summer Milan PM_10_ at a concentration of 2.5 *μ*g/cm^2^. CTRL: untreated cells. Results are the mean and s.d. of at least three independent experiments and presented as pg/mL released in the culture medium. ***P < 0.001 versus CTRL.

THP-1-derived macrophages were treated as reported with summer Milan PM_10_ and with winter PM_10_ and CB. The experiments showed that IL-1*β* was dose-dependently increased after summer Milan PM_10_ treatment, with a progressive increase from 1 to 5 *μ*g/cm^2^. In contrast, winter Milan PM_10_ and CB did not induce significant release of the interleukin (Figure 1). Cells were then exposed to 2.5 *μ*g/cm^2^ of summer Milan PM_10_, chosen as the first dose of effects, to investigate the time-course release of IL-1*β*. The IL-1*β* release showed a progressive increase from 2 to 6 h (Figure 2).

### 3.2. IL-1*β* Release and Inhibition of Caspase-1, TLR-2/-4 and Oxidative Stress

**Figure 3:**
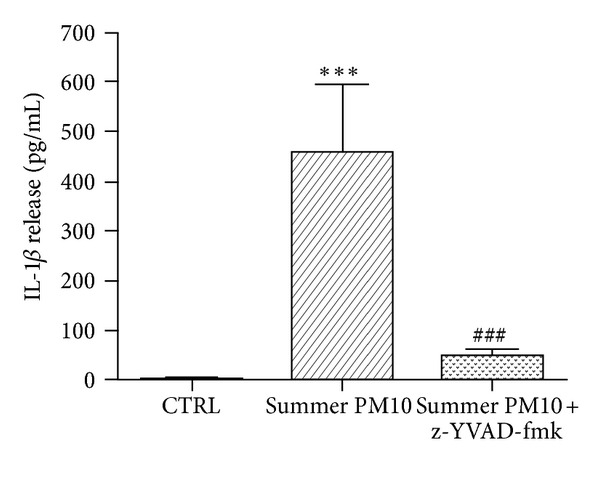
PM_10_-induced IL-1*β* release dependent on caspase-1 activation in THP-1-derived macrophages cells. The cells were pretreated with the caspase-1 inhibitor, z-YVAD-fmk (10 *μ*M) for 1 h, and exposed to summer PM_10_ for 6 h. Results are the mean and s.d. of three independent experiments. CTRL: untreated cells. ***P < 0.001 versus CTRL, ^###^ *P* < 0.001 versus summer PM_10_.

THP-1-derived macrophages were pre-treated for 1 h with the caspase-1 inhibitor z-YVAD (10 *μ*M) and then treated with summer PM_10_ (2.5 *μ*g/cm^2^) for 6 h, or only with z-YVAD. Cells preexposed with z-YVAD showed IL-1*β* release similar to the control (data not shown). The experiments showed that z-YVAD significantly reduced summer PM_10_-induced release of IL-1*β* (approximately 90%), compared to PM_10_ treatment alone (Figure 3).

**Figure 4:**
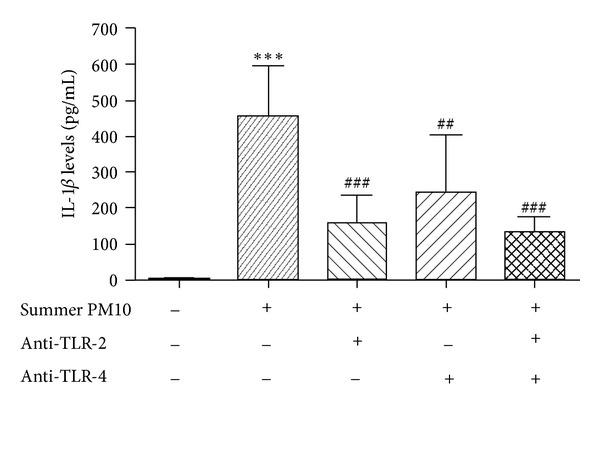
Inhibition of PM_10_-induced IL-1*β* release in THP-1-derived macrophages by TLR-2 and TLR-4 antagonist molecules. The cells were pretreated with TLR-2 and TLR-4 inhibitors (0.1 *μ*g/mL) for 1 h and then incubated in the presence (+) or absence (−) of summer PM_10_ (2.5 *μ*g/cm^2^) for 6 h. Results are the mean and s.d. of three independent experiments. ***P < 0.001 versus CTRL, ^###^ *P* < 0.001 versus summer PM_10_, and ^##^ *P* < 0.01 versus summer PM_10_.

Subsequently THP-1-derived macrophages were pretreated with TLR-2 and TLR-4 inhibitors (0.1 *μ*g/mL) for 1 h before exposure to summer PM_10_ (2.5 *μ*g/cm^2^ for 6 h). The inhibition of the TLR receptors significantly reduced the release of IL-1*β* induced by summer Milan PM_10_ (Figure 4). The TLR-2 inhibitor was more potent than the TLR-4 inhibitor. Furthermore, combining the two inhibitors gave the maximal reduction of IL-1*β* release. The TLR inhibitors did not affect the IL-1*β* release from control cells (data not shown).

**Figure 5:**
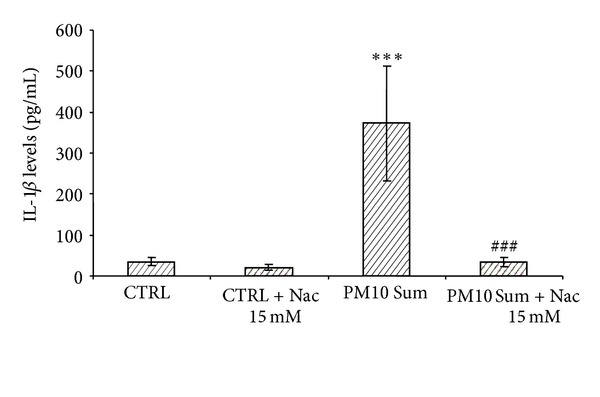
Inhibition of PM_10_-induced IL-1*β* release in THP-1-derived macrophages by NAC. The cells were pretreated with 15 mM NAC (+) and then incubated with summer PM_10_ (2.5 *μ*g/cm^2^) for 6 h. Results are the mean and s.d. of at least three independent experiments. ***P < 0.001 versus CTRL, ^###^ *P* < 0.001 versus summer Milan PM_10_.

Treatment with NAC, an inhibitor of oxidative stress, reduced the release of IL-1*β* in THP-1-derived macrophages treated with PM_10_ to control levels (Figure 5).

### 3.3. Cells-Particles Interaction: Endocytosis Pathway

We focused our study also on the molecular mechanisms involved in summer Milan PM_10_-induced IL-1*β* release. 

**Figure 6:**
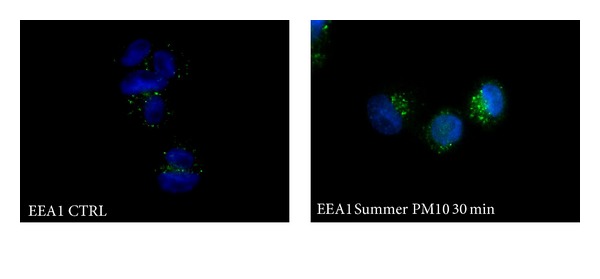
PM_10_-induced overexpression of early endosomes in THP-1-derived macrophages. The cells were treated with summer Milan PM_10_ at the concentration of 2.5 *μ*g/cm^2^ for 30 min and then stained for the early endosome antigen EEA1 (green). Nuclei are stained with DAPI (blue): representative images.

**Figure 7:**
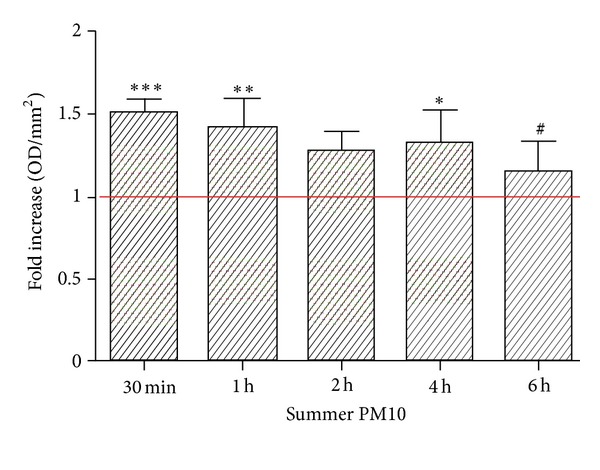
Early endosome antigen 1 EEA1 protein expression in THP-1-derived macrophages exposed to summer Milan PM_10_. The cells were treated with summer PM_10_ (2.5 *μ*g/cm^2^) for 30 min, 1 h, 2 h, 4 h, and 6 h and then assessed for EEA1 expression by Western analysis. Mean and s.d. of at least three independent experiments. ***P < 0.001 versus CTRL, **P < 0.01 versus control, *P < 0.05 versus CTRL, and ^#^ *P* < 0.05 versus 30 min.

The activity of early endosomes after the exposure to summer PM_10_ was examined by analysing the expression of the early endosome antigen 1 (EEA1) in THP-1-derived macrophages. The cells were exposed to summer PM_10_ for 30 min and then assessed by EEA1 immunostaining. We observed that EEA1 expression after 30 min was remarkably increased compared to the control (Figure 6). This result is also confirmed by immunoblotting of EEA1. The cells were treated with summer PM_10_ (2.5 *μ*g/cm^2^) for 2 to 6 h and assessed for EEA1 expression by Western analysis. The data show that the EEA1 is overexpressed from 30 min to 4 h, but was approximately similar to the control at 6 h (Figure 7). These results suggest that THP-1-derived macrophages are able to phagocytise summer PM_10_ and its internalization involves early endosomes.

### 3.4. Cell Particles Interaction

**Figure 8:**
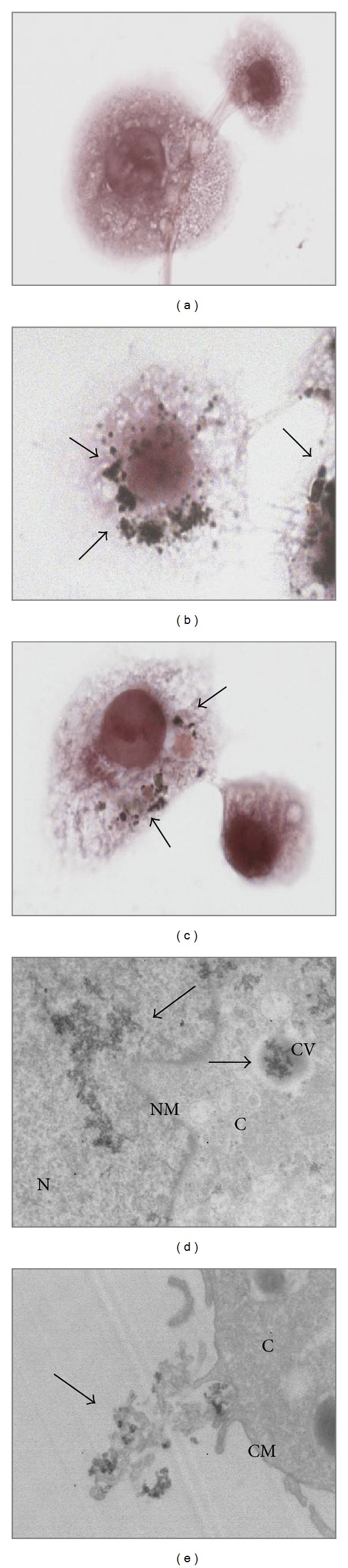
PM_10_ interaction with THP-1-derived macrophages. The cells were exposed to summer PM_10_ at the concentration of 2.5 *μ*g/cm^2^ for 4 h (b) and 6 h (c) and stained by haematoxylin eosin and compared with the control (a). An increased number of particles associated with the cells is indicated by black arrows. TEM images of the cells exposed to summer PM_10_ after 6 h are showed in (d, e), showing particles internalised in cytoplasm vesicles as well as particles into the nucleus (d). Particles interaction with the cell membrane are presented in (e). N: nucleus; NM: nuclear membrane; C: cytoplasm; CV: cytoplasmic vesicle; CM: cellular membrane. Magnification = 10 K.

The interaction between summer Milan PM_10_ and THP-1-derived macrophages was determined by haematoxylin-eosin staining and electron transmission microscopy (TEM). Haematoxylin-eosin-stained macrophages exposed to summer PM_10_ (for 6 h) showed a high number of particles attached to the cells (Figures 8(b) and 8(c)). TEM picture demonstrated the internalization of summer PM_10_ particles in cytoplasm vesicles and also translocation of small aggregates in the nucleus (Figure 8(d)). The TEM picture showed also a clear interaction of summer PM_10_ with plasma membranes with the formation of phagocyte structures (Figure 8(e)).

## 4. Discussion

Recently PM_10_ was demonstrated to induce IL-1*β* release via an inflammasome mechanism as revealed by caspase-1 inhibition and siRNA against NALP3 in THP-1 cells and by NALP3 knockout mice. The potential of PM_10_ was however relatively slight, about 3-fold using 500 *μ*g/mL [35]. Compared to this we report that PM_10_ collected in the summer in Milan induced a massive IL-1*β* response (a 50-fold increase, at 10-fold lower concentrations) in THP-1-derived macrophages. However, PM_10_ collected in Milan in the winter showed only a slight IL-1*β* response, underlining the importance of the PM_10_ sources. Our study also indicates the importance of an inflammasome mechanism, as the response was reduced by caspase- 1 inhibition. It is however suggested that the summer Milan PM_10_-induced activation of TLR-2 and TLR-4 receptors, leading to synthesis of pro-IL-1*β*, is the major determinant for the massive response induced by summer Milan PM_10_. Furthermore, our study showed a role for oxidative stress in the PM_10_-induced IL-1*β* release.

Most of the in vitro studies of particle-induced IL-1*β* responses have primed the cells with the endotoxins like LPS to increase the pool of pro-IL-1*β* before exposing to different agents capable of inducing the inflammasome mechanism. Indeed, for both crystalline and amorphous silica particles [20, 30], the priming of exposed cells with bacterial LPS is essential for the release of IL-1*β* following the activation of the inflammasome. It is now emerging in a lot of studies that different nanoparticles and other agents might activate the inflammasome mechanism, and induce large IL-1*β* responses in LPS-primed cells [30, 41]. In the present study it is also shown that the activation of the inflammasome is a crucial mechanism for the PM_10_-induced IL-1*β* response, as the caspase-1 inhibitor z-YVAD reduced the interleukin release. Furthermore, PM_10_ induced a marked increase in endosomes internalization that has been shown to be involved in the inflammasome pathway [22, 42]. The most striking in this study is however that the marked IL-1*β* response is observed in the absence of LPS-priming. This suggest that summer Milan PM_10_ contains constituents (endotoxins capable of the activation of TLR-2 and/or TLR-4) which lead to pro-IL-1*β* formation; in support of this, the suppression of TLRs activation by pretreating the cells with anti-TLR-2 and TLR-4 molecules induced a significant reduction of IL-1*β* release. Both the TLR-2 and 4 are receptors which role in recognition and binding of LPS, and other bacterial PAMPs have been extensively described [43, 44]. Since airways macrophages seldom recognize bacterial components individually, the response to different bacterial PAMPs is usually orchestrated by a combination of different TLRs. In fact we demonstrated that the combination of the two inhibitors seems to give the maximal reduction of IL-1*β* release after PM_10_ exposure. However, our data may indicate that other biological components or PAMPs and/or other membrane receptors may be involved in the priming of cells, since the reduction of TLR-2 and 4 activities did not abolish completely the final release of IL-1*β*. Indeed is has been reported that the activation of the different TRLs might be promoted by different molecules which can be found in PM [45]. The inhibition of TLR2 and 4 indicates the importance of these two receptors in summer Milan PM_10_-related effects, but the involvement of other TLRs cannot be excluded. Furthermore we have already reported [41] the presence of crustal elements in summer Milan PM_10_ which can promote proinflammatory responses. 

Also, in the study of Hirota [35], a PM_10_-induced release of IL-1*β* is observed without LPS priming, but to a much less extent than for PM_10_ collected in Milan in the summer. In accordance with Hirota, we found a modest IL-1*β* response upon treatment to winter Milan PM_10_. The differential effects can presumably be attributed to the content of endotoxins in the PM_10_. In fact, we have previously reported that coarse fraction of summer PM_10_ is rich in Gram-negative bacteria, expressing LPS, in addition to crustal elements among which also silica [39, 46]. Interestingly, with respect to other end-points, like DNA damage and apoptotic cell death, we have shown that winter Milan PM_10_ is more potent than summer Milan PM_10_.

Oxidative stress seems to be crucial for the PM_10_-induced IL-1*β* response, as demonstrated by inhibition by the inhibitor NAC. In addition to generation of ROS by a cell-free mechanism, ROS may be generated via the mitochondrial pathway [47]. Upon rupturing the lysosome membrane, the ROS may also be released to the cytoplasm [21]. A critical question is whether the ROS exerts its effect on IL-1*β* release after PM_10_ treatment by affecting the lysosome pathway or the pathway from TLR activation to pro-IL-1*β* formation. The increased number of endosomes subsequent to PM_10_ exposure reported in this study could indicate a release of ROS via this pathway that may be linked to inflammasome mechanism. However, ROS scavengers, such as NAC, have been reported to interact more with the priming of the NLRP3 rather than its activation [48]. A better understanding of the importance of ROS in the TLR-activation, subsequent NF-kB activation, and synthesis of pro-IL-1*β*, versus activation of the inflammasome, is thus needed.

### 4.1. Concluding Remarks

Since IL-1*β* has been related to a number of human diseases, including different pulmonary pathologies [49], and PM is known to increase the development of lung disease [50], it is crucial to increase the understanding of the pathways involved. Our findings, with very marked effect of PM_10_ in absence of exogenous addition of LPS, suggest that summer Milan PM_10_ contains constituents both related to the activation of membrane TLRs and activation of the inflammasome NLPR3. Furthermore, our study suggests that the activation of TLRs is of much importance for explaining the magnitude of the PM_10_-induced IL-1*β* response. Thus, PM_10_ containing less biological components (PM_10_ sampled in winter) induces only a minor IL-1*β* response. The present study indicates an important role for ROS in PM_10_-induced IL-1*β* formation, but further investigations are needed to elucidate the origin of the ROS (by lysosomes rupture or other pathways), and to what extent the effect is mediated by inhibition of the NLP3 activation or the TLR-pro-IL-1*β* pathway, or a combination of both these pathways.
